# Designing a bilingual health prevention website: challenges of using a participatory approach

**DOI:** 10.1093/eurpub/ckac129.641

**Published:** 2022-10-25

**Authors:** S Jaroof, H Cordel, B Badoro, B Felix, M Rolland, C Bourovali-Zade, J Cailhol

**Affiliations:** 1 Infectious Diseases Department, Paris Seine Saint Denis University Hospital, Bobigny, France; 2 Laboratoire Education et Promotion de la Sant, Sorbonne Paris Nord University, Bobigny, France; 3 Institut Convergences Migrations, ICM, Aubervilliers, France; 4 Centre régional de Prévention en Santé, CRIPS, Pantin, France; 5 Paris, France

## Abstract

**Issue/problem:**

For some migrants, specific health information tools are needed in order to counterbalance lack of access to information both in countries of origin and of destination.

**Description of the problem:**

Migrants originating from Pakistan and newly arrived in France are highly affected by for instance hepatitis C. Many of them have a low level of litteracy, including health litteracy. We decided to create a bilingual Urdu/French website, to provide information on hepatitis C, but also on sexual and mental health. The project was conceptualized in a participary manner, in order to fit the targeted population needs. The project started in June 2021 and the website is expected to be launched by September 2022. We report here some of the key challenges which emerged when working with such hard-to-reach community.

**Results:**

The participatory approach involving community members consisted of 3 steps. In step 1, we confirmed content needs, and assessed preferences in terms of type of media to be used and media styles. About 30 themes were prioritized and the preferred media was video with formal style. After writing scripts, we organized focus-groups discussions to create culturally-appropriate messages. A third and last step will be the selection of titles and keywords for internet search, after shooting videos.

**Lessons:**

Despite working closely with a Pakistani cultural association and urdu-speaking health professionnals, recruitment of community members for participating in the website design happened to be extremely difficult. Despite initial enthusiasm, interests in the project tended to decrease according to sometimes hidden motivations. Focus groups dynamics were at times affected by significant differences in social situations between participants. Despite these challenges, the participatory process allowed us to reshape part of the content according to the community's communications codes, in order to enhance the efficiency of the messages.

**Key messages:**

• Working with socially disadvantaged community members is crucial in order to reduce social inequalities in health.

• However, caution is needed to anticipate and decrease unfulfilled expectations.

